# Engineering the control of mosquito-borne infectious diseases

**DOI:** 10.1186/s13059-014-0535-7

**Published:** 2014-11-15

**Authors:** Paolo Gabrieli, Andrea Smidler, Flaminia Catteruccia

**Affiliations:** University of Pavia, Pavia, 27100 Italy; Department of Immunology and Infectious Diseases, Harvard School of Public Health, Avenue Louis Pasteur, Boston, MA 021155 USA; Department of Genetics, Harvard Medical School, Avenue Louis Pasteur, Boston, MA 02115 USA; Department of Microbiology, Perugia University, Perugia, 06100 Italy

## Abstract

Recent advances in genetic engineering are bringing new promise for controlling mosquito populations that transmit deadly pathogens. Here we discuss past and current efforts to engineer mosquito strains that are refractory to disease transmission or are suitable for suppressing wild disease-transmitting populations.

## Introduction

Mosquitoes transmit a variety of infectious agents that are a scourge on humanity. Malaria, dengue fever, yellow fever, and other mosquito-borne infectious diseases infect millions of people and account for hundreds of thousands of deaths each year, posing a huge burden for public health and on the economic growth of countries where these diseases are endemic [1]. Given the lack of effective vaccines against many mosquito-borne pathogens, national programs are heavily reliant on the use of insecticides to control mosquito populations in order to stop disease transmission [2]. Unfortunately, the alarming pace of emergence of insecticide resistance in mosquitoes [3] is threatening chemical-based campaigns and is forcing scientists to develop alternative strategies to combat vector-borne diseases. Moreover, insecticide-treated bed nets and indoor residual sprays principally target mosquitoes that feed indoors at night and that rest inside houses, thereby neglecting those species that prefer to bite and rest outdoors or at earlier hours of the day, and inducing some degree of insecticide-avoidance behavior (behavioral resistance) in indoor-biting individuals [4-6].

**Figure 1:**
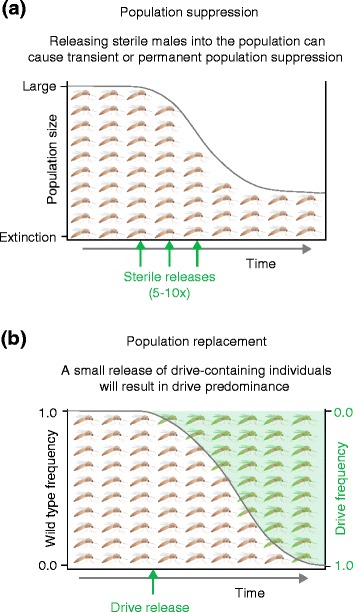
**Methods for the genetic control of vector populations. (a)** Population suppression can be achieved by releasing large numbers of males that render their wild female mates incapable of having viable progeny. This includes releasing either males that are sterile and produce no progeny at all (as in sterile insect technique (SIT)) [15] or males that pass on lethal transgenes to the next generation, producing progeny that die before they can transmit disease (as in the release of insects carrying dominant lethals, RIDL) [16]. For SIT strategies, multiple releases of a large excess (5x to 10x) of sterile males relative to the target population are normally carried out over large areas. **(b)** Population replacement occurs when traits carried by a small number of engineered mosquitoes replace traits that naturally exist in field populations [17]. The desired engineered trait - for instance, an anti-pathogen gene that renders mosquitoes refractory to disease transmission - is driven to fixation in the field population using a genetic drive (as described in Figure 2h).

Recent major advances in the field of genetic engineering are providing an unprecedented opportunity to conceive and create designer mosquito strains in order to control natural vector populations. From the generation of the first transgenic mosquitoes [7-10] to the creation of the first gene knock-outs [11-13], the discovery of genetic tools has revolutionized our ability to functionally study and edit the mosquito genome. In the fight against infectious diseases, vector populations can be modified using these tools in two principal ways: 1) they can be made refractory to disease transmission by the introduction of genes with anti-pathogenic properties; 2) they can be rendered sterile or modified in such ways that the population size will crash below the threshold necessary to support disease transmission (Figure 1) [14]. Both strategies have strengths and limitations that are inherent to their design and properties.

Genetic engineering technologies include those that allow heterologous gene expression and those that modify endogenous genes or entire portions of the mosquito genome. Here we review the genetic tools that are currently in use and those that promise to become available in the near future, with particular focus on those techniques that are capable of reprogramming the genomes of field populations. We also discuss current field trials in which genetically modified mosquitoes are being released, and will mention ecological hurdles and potential environmental and regulatory issues stemming from the release of genetically modified insects into the wild.

## First generation of anti-pathogenic strains

**Figure 2:**
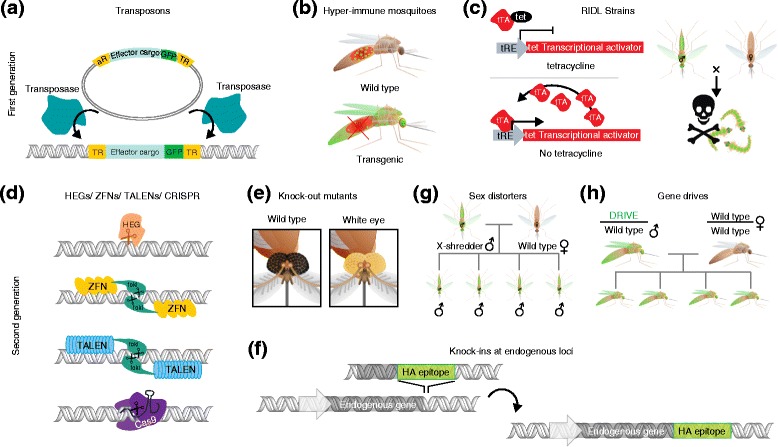
**Current and future genetic engineering technologies for vector control. (a)** First-generation technologies make use of transposable elements to insert genetic cargo randomly into the genome. The transposable element is mobilized by a transposase enzyme produced by another plasmid, which recognizes and cleaves the terminal repeats (TR) of the transposon cassette and mediates insertion of the transposable element into the genome. Insertion is visualized using selectable markers such as the green fluorescent protein (GFP) [19]. **(b)** Mosquitoes can be engineered to carry anti-pathogenic effector genes that reduce the pathogen load [21-31]. In the figure, the effector gene blocks *Plasmodium* ookinete invasion of the midgut epithelium, preventing oocyst development. **(c)** Schematic of the RIDL system currently used for suppression of *Aedes aegypti* populations [16]. In the presence of tetracycline, expression of the tetracycline transactivator (tTA) is repressed. In the absence of tetracycline, tTA binds to the tetracycline-responsive element (tRE) and drives its own expression in a positive feedback loop that leads to the accumulation of toxic levels of tTA. The progeny of released males carrying this transgene are not viable. Other combinations of inducible systems and toxic genes can be used in place of tTA and tRE to achieve population suppression. **(d)** Second generation technologies include HEGs, ZFNs, TALENs and CRISPR/Cas9 [11-13,32,33]. These technologies facilitate double-stranded DNA breaks in the genome at desired loci. **(e)** HEGs, TALENs and ZFNs have been used in *Ae. aegypti* and *Anopheles gambiae* to generate null mutants [11-13], including eye color mutants [11]. **(f)** ZFNs have been used to generate site-specific knock-ins of exogenous sequences in *Ae. aegypti* [34]. The figure illustrates a possible application for knock-in technology, which would enable scientists to fuse protein domains to the end of endogenous genes. These domains include those encoding fluorescent proteins or epitope tags, such as an HA tag (shown). **(g)** Sex distorter strains make use of an HEG, I-PpoI, to destroy sperm carrying an X chromosome (X-shredder), producing male-only populations. When mated to wild-type females, transgenic males sire only sons, potentially leading to population suppression [35]. **(h)** Gene drives are genetic elements that are inherited in a non-Mendelian fashion and can spread through populations. Gene drives using HEGs have been successfully developed to drive through laboratory mosquito populations [36], whereas evolutionarily stable drives enabled by CRISPR/Cas9 have been proposed [37].

The expression of exogenous genes - through the transposon-mediated integration of transgenes - was the first genomic technology to be developed in mosquitoes, and gave birth to the modern field of mosquito genome engineering [7-10]. In this initial system, different exogenous ‘effector’ genetic elements are cloned between the transposon terminal repeats (usually using the *PiggyBac* transposon [10]) to form a synthetic element that, in the presence of the integrating enzyme transposase, inserts into the mosquito genome at quasi-random loci (Figure 2a). In order to identify successful transformants, synthetic transposons are generally designed to carry a fluorescent reporter construct, such as the green fluorescent protein (GFP), that acts as a selectable marker [18]. The promoter of choice for the expression of selectable markers is often the neuronal 3xP3 promoter [19], which is expressed during larval development allowing easy detection of fluorescence and facilitating high-throughput sorting by automated live sorters [20]. Moreover, this system can incorporate cargoes with anti-pathogenic properties to render mosquitoes refractory to disease transmission.

Both *Anopheles* and *Aedes* mosquito species, the vectors of malaria and dengue, respectively, have been modified to reduce their vectorial capacity. To stop the development of *Plasmodium* parasites, the causative agents of malaria, scientists have developed transgenic *Anopheles stephensi* lines that express single chain variable fragment antibodies (scFvs) [21-23] or synthetic antimalarial factors [24,25] (Figure 2b). Transgenic lines that express ScFvs against the ookinete proteins Chitinase 1 and Pfs25 [38,39] or the predominant surface protein of the sporozoites, circumsporozoite protein [40,41], show reduced ookinete crossing of midgut walls or sporozoite invasion of the salivary glands, respectively. Similarly, *An. stephensi* strains have been generated that secrete the synthetic dodecapeptide SM1 (an acronym for salivary gland- and midgut-binding peptide 1) into the midgut lumen during blood feeding. SM1 binding to the epithelium - probably through a mosquito midgut receptor - prevents ookinetes from invading the midgut in the rodent malaria *Plasmodium berghei* model, thereby reducing both the prevalence and the intensity of infection [24]. Additionally, the incorporation of bee venom phospholipase A2 into transgenic *An. stephensi* inhibits ookinete invasion of the midgut by modifying epithelial membranes [25]. *Anopheles gambiae*, the principal vector of malaria in sub-Saharan Africa, has been engineered to ectopically express the endogenous antimicrobial peptide cecropin A [26] and the synthetic peptide Vida3 [27], a hybrid peptide based on natural antimicrobial peptide sequences that have strong activity against *Plasmodium* sporogonic forms [28].

Different laboratories have also developed *Anopheles* strains modified in key endogenous cellular pathways that regulate parasite development, namely the insulin-growth factor signaling (ISS) and the immune deficiency (IMD) pathways. In *An. stephensi*, overexpression of Akt, a critical regulator of ISS, elicits mitochondrial dysfunction that enhances parasite killing in the midgut, even if at some cost to mosquito survival [42,43]. To overcome fitness costs, an inhibitor of ISS, the phosphatase and tensin homolog (PTEN), was instead overexpressed [44]. PTEN inhibits phosphorylation of the ISS protein FOXO, and its expression blocks *Plasmodium* development by enhancing the integrity of the midgut barrier, although this causes an increase in the female lifespan with possible negative consequences for disease transmission [44]. In another study, *An. stephensi* mosquitoes were engineered to express the active form of the IMD-regulated NF-κB transcription factor Rel2-S. Rel2-S activates the expression of several antimicrobial and anti-*Plasmodium* peptides, and when overexpressed in the midgut and in the fat body, it strongly inhibits parasite development [45].

Engineering pathogen resistance has not been limited to anophelines. Dengue virus infections in *Aedes aegypti* mosquitoes have been attenuated by exploiting the natural antiviral RNA interference pathway. An inverted-repeat RNA capable of forming double-stranded RNAs that target the pre-membrane protein coding region of the DENV-2 serotype was expressed in the midgut [29] or in the salivary glands [30]. This modification reduced viral titers by more than five-fold compared to those in control mosquitoes. It should be noted, however, that multiple dengue serotypes (as well as multiple human malaria parasites) exist, complicating population replacement efforts aimed at spreading pathogen-refractory genes into wild populations.

## First generation of sterile strains for population suppression

Early transposon-based technology has been also used to generate mosquito strains aimed at the suppression or elimination of vector populations through the release of sterile males (the sterile insect technique (SIT)) [15]. The alternative sister strategy is the release of insects carrying a dominant lethal (RIDL) modification [16]. SIT is based on the release of large numbers of sterile males, usually sterilized with high doses of irradiation or chemical sterilants, that upon mating with field females produce no fertile progeny causing suppression or elimination of local populations (Figure 1a) [15]. The sterilization process usually induces severe fitness costs in the male, such that larger numbers of males than those initially predicted by simple models need to be released to achieve the desired level of suppression [46]. Genetic engineering can not only enable high-throughput sorting of male-only populations based on sex-specific fluorescent markers [47,48], but can also enable the design of strains in which specific sterility-inducing transgenes or genetic mutations have been introduced without causing the fitness costs associated with irradiation [49,50]. The most successful RIDL example is provided by the *Ae. aegypti* strain OX513A [16], which carries an inducible dominant genetic system that kills late larval stages. This system is composed of a gene encoding the tetracycline transactivator (tTA) protein under the control of the tetracycline-responsive element (tRE). Binding of tetracycline to tTA prevents tTA from activating transcription; when tetracycline is removed, tTA instead binds to tRE, thereby inducing its own expression via a positive feedback loop. The accumulation of tTA is toxic to cells and ultimately leads to organismal death (Figure 2c). This repressible system allows the generation of males that are fertile in the laboratory but that, once released, sire unviable progeny upon mating with field females. These RIDL strains are already being released in different geographical locations as part of field trials.

A different approach, initially developed in *Ae. aegypti* and now transferred to *Aedes albopictus* and *An. stephensi*, is based on a bimodular system that severely impairs the functionality of the female flight muscles, disrupting the female’s ability to fly (fsRIDL) [51-53]. The first module consists of tTA under the control of the female-specific *Actin-4* transcriptional regulatory elements, which drive gene expression in the indirect flight muscles of female pupae. The second module comprises a lethal gene (*Nipp1Dm* or *michelob_x* in *Ae. aegypti*, *VP16* in *Ae. albopictus* and *Nipp1Dm* in *An. stephensi*) under the control of tRE. In the absence of tetracycline, expression of the lethal gene specifically in the female flight muscles causes cell death and inability to fly. As males are unaffected by the transgene, their release will generate flightless female progeny that are unable to mate, bite, and transmit disease, eventually leading to population suppression [51].

## Second generation transgenesis provides increased flexibility

New genome-editing tools now allow scientists to modify endogenous genes with increasing flexibility and ease, and are being utilized in the laboratory with promising results to reduce the vectorial capacity of mosquito vectors (Figure 2d). The flexibility of these tools resides in the use of protein precursors that can be designed to bind sequences of interest within the mosquito genome [11-13]. Repetitive zinc finger (ZF) and transcription activator-like effector (TALE) modules have been successfully fused to the endonucleolytic domains of a type II endonuclease, normally FokI, to generate knock-out and knock-in mutants [11-13,34] (Figure 2e,f). These modified nucleases cause site-specific double-stranded DNA breaks that can be repaired by the non-homologous end-joining (NHEJ) pathway, an error-prone repair pathway that often results in small indels. As a basic proof-of-principle, this technology has been used to generate eye-color mutants (Figure 2e) [11], but it can also help elucidate pathways that are important for vector competence. For example, TALE nucleases (TALENs) have been used in *An. gambiae* to generate null mutants of the *thioester-containing protein 1* (*TEP1*) gene, a complement-like factor that opsonizes *Plasmodium* parasites in the midgut and mediates their killing. Mutant strains are, therefore, hyper-susceptible to *Plasmodium* infection [13], and although not directly employable for malaria control, they allow detailed genetic analyses of anti-*Plasmodium* immune pathways. Similarly, the zinc-finger nuclease (ZFN)-mediated knock-out of the odorant receptor co-receptor (ORCO) in *Ae. aegypti* has enabled the analysis of pathways involved in host-seeking behavior for blood feeding [12], opening up new avenues for the development of mosquito repellents and attractants. In another study, the CO_2_ response of *Ae. aegypti* mosquitoes was analyzed in mutants that have a defect in the *AaegGr3* gene, which encodes a subunit of the heteromeric CO_2_ receptor, contributing to our understanding of mosquito attraction to humans [34]. This mutant, the first knock-in to be reported in mosquitoes, was generated by the disruptive insertion of a fluorescent reporter gene into the *AeagGr3* locus. Such knock-in technology could also be used to facilitate in-frame insertions of protein tags into genes of interest, further enabling the study of complex pathways in mosquitoes (Figure 2f).

Homing endonucleases (HEGs) have also been successfully used to manipulate the mosquito genome [32,54,55]. HEGs are double-stranded DNases targeting large (12 to 40 bp) asymmetric recognition sites that occur extremely rarely in genomes [56]. *An. gambiae* strains have been generated that express I-*Ppo*I, a HEG that recognizes and cuts a site in a multi-copy rDNA gene, which in this species is located exclusively on the X chromosome [35,57]. When I*-Ppo*I is expressed specifically during spermatogenesis, it cleaves these multiple target sequences causing shredding of the paternal X chromosomes in sperm cells [35,57]. This feature was originally meant to generate male-only populations by preventing fathers from transmitting the X chromosome to embryos; but I-*Ppo*I expression in sperm cells induces complete embryonic lethality, probably as a consequence of the shredding of the maternal X chromosome upon unintended transfer of the enzyme to the embryo [57]. These strains induce a high level of infertility in large cage trials, as discussed below [58]. An improved version of these strains, which carries a less thermostable version of I-PpoI with reduced *in vivo* half life, has been generated that is instead active only in the testes, causing the specific shredding of the paternal X chromosome in sperm without directly affecting the embryo [35] (Figure 2g). The resulting sex-distorter strains produce >95% male offspring and are able to suppress wild-type mosquito populations in laboratory cages [35].

## Gene drives for population replacement

For the implementation of population replacement strategies aimed at curbing mosquito-borne diseases, the anti-pathogen constructs described above need to be driven genetically through natural populations so that the disease refractory traits will spread (Figure 2h). A number of artificial gene-drive systems capable of forcing their own spread in a non-Mendelian manner are being developed that could be used for this purpose. In the model organism *Drosophila melanogaster*, the first gene-drive mechanism was developed on the basis of a toxin-antidote system [59]. This synthetic system, named *Medea* after the mythological figure of the woman who killed her own children to take revenge on her husband’s betrayal, is based on expression in the zygote of a toxic gene, such as a microRNA against a maternal mRNA essential for embryonic development [59,60]. Transgenic females carry an ‘antidote’ , that is, an allele of the gene that is insensitive to the toxin, allowing transgenic progeny to survive and spread the transgene. Although *Medea* has yet to be adapted to disease vectors, HEG-based technologies have been suggested and tested as gene drives in mosquitoes [36,61]. In this system, the drive encodes DNA-cutting machinery that cleaves a wild-type target locus from a transgene located at the homologous locus. Repair of the DNA break by homologous recombination causes the transgene to copy into the cleaved locus, causing a hemizygous cell to become homozygous for the transgene (Figure 2h). If this mechanism occurs in the germline, the transgene can spread through the population, potentially carrying an anti-pathogenic construct with it. Proof-of-principle use of HEGs to facilitate gene-drive mechanisms in *An. gambiae* was based on the I-SceI enzyme, which targeted its own recognition sequence that had been artificially introduced into a *GFP* reporter gene [36]. Homing of the HEG into its target sequence, previously integrated into the mosquito genome, would therefore generate GFP null mutants. Small cage experiments indicated that I-SceI could rapidly invade the receptive target strain, providing the first evidence of the gene-drive capabilities of HEGs in mosquitoes [36].

The range of applications enabled by HEGs and other nuclease-based technologies (ZFNs and TALENs) has some limitations, especially in terms of specificity, flexibility and stability. For example, ZFNs do not always have the desired sequence specificity when assembled into arrays, which limits the number of loci that can be targeted [62]. HEGs have been shown to cleave non-target sites (for a review see [63]), and laborious *in vitro* studies are necessary to generate new enzymes that have the required sequence specificity [64]. Furthermore, as these systems cut a single genomic sequence at a time, new transgenic strains must be created for each target sequence. A new genome-engineering tool, CRISPR/Cas9 (for clustered regularly interspaced short palindromic repeats/CRISPR-associated protein 9), has the potential to overcome these limitations and stimulate the generation of effective gene drives for vector control. Discovered as the molecular machinery of a bacterial acquired immune defense system [65], CRISPR/Cas9 was soon co-opted to engineer the genomes of a wide variety of organisms with high flexibility and efficiency [33]. Cas9 is an endonucleolytic protein that can recognize and cleave specific genomic sequences with the help of a small artificial guide RNA (gRNA). When the gRNA and Cas9 form a complex, they catalyze DNA cleavage upon recognition of the target site by the gRNA. The reliance on easily designed gRNAs for the recognition of target sequences results in a significant increase in the number of genomic loci that can be cleaved when compared to other systems, as RNA-guided engineering does not require modification of the Cas9 protein itself. Moreover, a number of loci can be targeted simultaneously by providing multiple gRNAs, thereby reducing the possible emergence of resistance to cleavage [37]. Although research demonstrating the use of CRISPR/Cas9 in mosquitoes has yet to be published, it is likely that this technology will soon enable the development of innovative and evolutionarily stable gene drives for the control of vector-borne diseases. Nevertheless, further research is needed to demonstrate the improved performance of this system over already existing technology, including minimizing off-target cleavage events and the possibility to revert the effects of the introduced gene architectures [37].

## Current field trials utilizing genetically modified mosquitoes to fight disease

Intensive research is ongoing to generate improved engineered strains that are suitable for vector-control programs, but the first generation of genetically modified mosquitoes is already being released in the field. Since 2009, the UK-based biotech company Oxitec has been pushing the boundaries of genetic control by operating the first releases of transgenic *Ae. aegypti* RIDL strains to suppress wild populations [66-69]. Their aim is to test the efficacy of these strains as a tool against dengue, a viral disease for which no vaccine or effective drugs are available. Repeated releases of the RIDL strain OX513A achieved a sizable reduction of wild populations, bringing new promise for disease control. The first program was operated on Grand Cayman Island, a British Territory in the Caribbean [66]. An average of 465 males/hectare (ha)/week were released across 10 hectares over a 4-week period, representing about 16% of the male population in the field. A total of 9.6% of fluorescent larvae were detected from eggs collected in ovitraps three weeks after the release, demonstrating that RIDL males could mate with wild females and sire progeny, despite their reduced field competitiveness. A subsequent program, using 3,500 males/ha/week, was carried out over a 23-week period and achieved 80% suppression of the wild population in a 16-ha area [67]. To accomplish this task, 3.3 million engineered males were reared and released, stressing the need to optimize mass-rearing protocols [69]. OX513A was also released in a forested area in Pahang, Malaysia, and transgenic males were shown to live as long as their wild-type brothers from the same laboratory strain, even if their dispersal ability was reduced [68]. Releases of OX513A are currently being performed in Brazil [69], where additional trials are planned and the mosquito production factory is being expanded. Large outdoor field cages have also been employed to test the potential use of the flightless *Ae. aegypti* fsRIDL strain [51,70]. This strain did not, however, achieve complete suppression of target populations, suggesting that it may not be suitable for large-scale releases [70]. Reduced mating competitiveness of transgenic males probably contributed to test failure but other explanations, including the different genetic backgrounds of released individuals and wild populations, have also been proposed [70].

In the case of malaria vectors, large caged laboratory trials have been established to test the mating competiveness of sterile *An. gambiae* males carrying the HEG I-PpoI. When released at 5- to 10-fold coverage in large cages, I-PpoI males induced high levels of infertility, leading to the suppression of caged populations in 4 to 5 weeks, despite showing reduced mating competitiveness [58]. Males carrying a less thermostable version of I-PpoI, which causes sex distortion rather than male infertility, also achieved elimination of caged populations within six generations when released at a 3x ratio [35]. Before the field release of these strains is contemplated, their competitive performance and sterilizing activity will need to be tested in semi-field settings, such as those provided by large outdoor enclosures, where mosquitoes are exposed to normal environmental conditions and must produce appropriate swarming and mating behavior [71].

## Ecological hurdles and environmental and regulatory considerations

**Figure 3:**
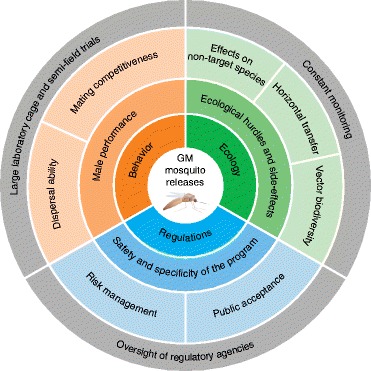
**Challenges for the field release of transgenic mosquitoes.** This scheme summarizes the ecological, behavioral and regulatory issues faced by disease control programs based on the release of genetically modified mosquitoes. Ecological requirements are shown in green, behavioral requirements in orange, while regulatory issues are presented in blue. Light-grey sections highlight operational tools that may be used to comply with the requirements. Behavioral requirements include key fitness parameters such as the dispersal ability and mating competitiveness of released males, and can be tested in large laboratory cage trials and then in semi-field settings to select the mosquito strains with the greatest probability of success. Ecological hurdles comprise heterogeneity in the genetics, behavior and natural habitats of vector species (biodiversity), and possible unintended side-effects on non-target species or on the ecosystem. Monitoring of these effects must be constantly in progress in the release phase. The risks, safety and specificity of the engineered strains need to be evaluated by appropriate regulatory agencies, and early public engagement is a priority.

The implementation of genetically modified mosquitoes in vector control programs is challenged by a number of ecological, environmental and regulatory issues (summarized in Figure 3). Two crucial behavioral components of the released males are dispersal ability, which affects the possibility of targeting populations in impenetrable regions [68], and mating competitiveness, especially for species with complex sexual behaviors [72]. Indeed, the mating fitness of released males has proven to be an important limiting factor in previous campaigns aimed at reducing the size of *Anopheles* populations (for a comprehensive discussion of these issues see [73] and references therein). Generally, anopheline species mate in elaborate swarms that are highly demanding energetically, and in which males are subject to strong competition to find a mate [74]. Reduction of competitiveness can be caused by a number of factors including but not limited to mass rearing, inbreeding, transposon expression and insertion sites in the genome [75-77]. The latter issue can now be partially overcome by utilizing ‘docking’ strains that are selected on the basis of limited fitness costs, using the PhiC31 integration system [78].

Other ecological features, including the biodiversity of native vector species, will also determine the success of a release campaign (Figure 3). Malaria transmission is supported by over 30 major primary vectors [79], many of which are morphologically indistinguishable [80]. These often sympatric species exhibit distinct behaviors in terms of mating, blood feeding and resting, and inhabit diverse ecological niches, making their control extremely arduous [81]. Such complexity represents a significant hurdle to the implementation of genetic engineering for malaria control; elimination of this disease solely by transgenic means would require the simultaneous release of all malaria-transmitting species in any given area, a highly arduous task. By contrast, dengue virus infections are transmitted worldwide principally by *Ae. aegypti* and few other *Aedes* species. Although genetic variations between different *Ae. aegypti* populations have been detected [82], pilot RIDL anti-dengue campaigns suggest that a single transgenic strain can adapt to different ecological contexts [67-69]. The same strain could potentially be deployed to reduce the spread of the other viral diseases transmitted by these mosquitoes, such as yellow fever and Chikungunya, the latter being an emerging threat in the Americas [83].

Finally, although the scope of this review is to describe the state of the art in transgenic technologies for disease control, we should mention that the release of genetically modified mosquitoes generates environmental and safety challenges that deserve to be meticulously addressed in each individual case (outlined in Figure 3). Unintended ecological side effects, accidental spread to non-target species, and horizontal transfer of the transgenes are all unlikely but possible negative scenarios that can and must be safely minimized [84]. Test trials under high containment levels and in confined laboratory and semi-field settings should be used to determine specificity and safety of modified vectors, and constant monitoring should occur during the release phase. This is especially important when releasing gene-drive architectures that are capable of spreading through entire populations, such as those afforded by meiotic drives, HEGs and CRISPRs. The fast and exciting pace of progress provided by genetic-engineering technologies requires an open and early discussion to engage regulatory agencies, the scientific community, and the public [85]. The end goal of genetic engineering for mosquito control is to provide future generations with the undisputable benefits of a world free of vector-borne pathogens, while ensuring that possible unanticipated ecological and environmental consequences are eliminated.

## Abbreviations

Cas9: CRISPR-associated protein 9
CRISPR: Clustered regularly interspaced short palindromic repeats
fsRIDL: Female-specific RIDL
GFP: Green fluorescent protein
gRNA: Guide RNA
HEG: Homing endonuclease
IMD: Immune deficiency pathway
ISS: Insulin-growth factor signaling
NHEJ: Non-homologous end-joining
ORCO: Odorant receptor co-receptor
PTEN: Phosphatase and tensin homolog
RIDL: Release of insects carrying a dominant lethal
scFv: Single chain variable fragment antibody
SIT: Sterile insect technique
SM1: Salivary gland- and midgut-binding peptide 1
TALE: Transcription activator-like effector
TALEN: Transcription activator-like effector nuclease
TEP1: Thioester-containing protein 1
tRE: Tetracycline-responsive element
tTA: Tetracycline transactivator
ZF: Zinc finger
ZFN: Zinc finger nucleases
